# Climate Change: A Major Factor in the Spread of *Aedes aegypti* (Diptera: Culicidae) and Its Associated Dengue Virus

**DOI:** 10.3390/insects16050513

**Published:** 2025-05-11

**Authors:** Shahid Majeed, Waseem Akram, Muhammad Sufyan, Asim Abbasi, Sidra Riaz, Shahla Faisal, Muhammad Binyameen, Muhammad I. Bashir, Shahzad Hassan, Saba Zafar, Oksana Kucher, Elena A. Piven, Olga D. Kucher

**Affiliations:** 1 Department of Entomology, University of Agriculture, Faisalabad 38040, Pakistanasimuaf95@gmail.com (A.A.); 2 District Head Quarter (DHQ), Faisalabad 38040, Pakistan; 3 Department of Statistics and Center of Data Science, Government College University, Faisalabad 38040, Pakistan; 4 Department of Entomology, Faculty of Agricultural Sciences & Technology, Bahauddin Zakariya University, Multan 60800, Pakistan; 5 Epidemic Prevention and Control Program, Directorate General Health Services Punjab, Lahore 54000, Pakistan; 6 Department of Biochemistry and Biotechnology, The Women University Multan, Multan 66000, Pakistan; 7 College of Naturopathic Medicine, East Grinstead, West Sussex RH19 4LZ, UK; 8 Public Health, Health and Hygiene, RUDN University, 6 Miklukho-Maklaya St., 117198 Moscow, Russia; 9 Department of Environmental Management, Institute of Environmental Engineering, RUDN University, 6 Miklukho-Maklaya St., 117198 Moscow, Russia

**Keywords:** climate, mosquitoes, dengue, meteorological variables, developmental period, population dynamics

## Abstract

Climate change is thought to be responsible for the spread of various vector-borne diseases. The current study was conducted to evaluate the impact of different temperature and relative humidity regimes on the developmental stages of the yellow fever mosquito, *Aedes aegypti* (Diptera: Culicidae). The study also evaluated the impact of larval density on the survival of *Ae. aegypti*. In addition, the association between vector larval abundance, dengue incidence, and climatic factors were elucidated during 2016–2019 in three populated districts of Punjab, Pakistan, i.e., Lahore, Rawalpindi, and Multan. The results of the study revealed that at 10 °C and 35 °C, egg hatching and adult emergence were significantly reduced, regardless of the relative humidity. In contrast, at 20 °C and 30 °C, the rates of egg and adult survival increased with higher relative humidity. In addition, a density-dependent response was observed regarding larval survival of *Ae. aegypti*. Moreover, larval incidence was positively correlated with the number of dengue patients, Tmax, RH, and precipitation at Lahore (0.55, 0.23, 0.29, and 0.13), Rawalpindi (0.90, 0.30, 0.21, and 0.14), and Multan (0.05, 0.27, and 0.13) respectively, except in Multan, where a negative correlation (−0.09) with precipitation was observed. The inflow of patients had a positive correlation with the occurrence of a larval population, relative humidity, and precipitation at Lahore, Rawalpindi, and Multan districts, with the scale values of 0.55, 0.25, and 0.16; 0.90, 0.22, and 0.03; and 0.05, 0.06, and 0.03, respectively. In addition, a forecast model, ARIMA, predicted that there was a higher rate of larval occurrence in Rawalpindi, followed by Lahore. This study concluded that the role of precipitation > 200 mm prior to a 1–2-month lag, a 20–30 °C temperature range, and an RH exceeding 60% lead to the occurrence of larvae and dengue case spikes. This study will help to reinforce dengue surveillance and control strategies in Pakistan and to establish early management strategies based on changing climatic factors.

## Simple Summary

This study investigates the impact of climatic factors on the development and survival of *Aedes aegypti*, the primary vector of dengue fever, in three major districts of Punjab, Pakistan—Lahore, Rawalpindi, and Multan—between 2016 and 2019. The findings indicated that extreme temperatures (10 °C and 35 °C) significantly reduced egg hatching and adult emergence, regardless of relative humidity. Conversely, at moderate temperatures (20 °C and 30 °C), a higher relative humidity increased survival rates. Moreover, larval survival was found to be density dependent. Statistical analyses revealed a positive correlation between mosquito abundance and climatic factors, including temperature, humidity, and precipitation, across all three districts. Moreover, dengue incidence exhibited a strong association with mosquito population levels, particularly following periods of heavy rainfall (>200 mm) with a 1–2-month lag. Forecasting using the ARIMA model predicted a higher mosquito population in Rawalpindi and Lahore compared to Multan. These findings emphasize the necessity of climate-based dengue surveillance and early intervention strategies to mitigate vector outbreaks.

## 1. Introduction

The climate is changing at an unprecedented rate due to global anthropogenic activities [1,2]. The global earth temperature has increased approximately 0.7 °C in the last century [3]. Climate change has a remarkable impact on the natural ecosystems [4], including insect vector dispersal, infestation, and the dynamics of infectious diseases [5,6]. Infectious diseases, i.e., dengue, Zika, yellow fever, malaria, etc., vectored by certain mosquito species play a significant role in human morbidity and mortality [7,8,9].

Abiotic factors, i.e., temperature, relative humidity, and precipitation, are the key components that regulate the behavior, life cycle, and population dynamics of insect vectors [10]. Moreover, insect vectors associated with arbovirus transmission, which replicate within the bodies of cold-blooded vectors, are highly dependent on the surrounding climatic temperature [11,12]. Temperature usually affects key life history parameters of insects, such as their wing size, blood-feeding potential, adult longevity, fecundity, gonotrophic cycle length, and the biting rate of mosquitoes. The fecundity of mosquitoes usually decreased significantly with an increase in temperature [13,14]. However, a higher temperature usually enhances the growth rates of disease-causing pathogens [15]. Warmer climates facilitate insect vectors, including female mosquitoes, to enhance their blood-feeding frequency, which increases pathogen transmission intensity [16], while a cold climate significantly limits the pathogen transmission capacity [17].

Insect vectors, including mosquitoes, are not geographically restricted, as many vector-borne diseases, including vivax malaria, West Nile fever, dengue fever, and chikungunya fever, recurred in Europe due to recent climatic variations [15,18]. Similarly, in Asia, the dengue epidemic geographically expanded from Southeast Asian countries to Sri Lanka, the Maldives, India, Pakistan, and China [14]. However, in Pakistan, the yellow fever mosquito, *Aedes aegypti* Linnaeus (Diptera: Culicidae), caused the first dengue epidemic in 1994 in Karachi city of Sindh province [16]. Again, since 2010, the dengue epidemic spread sporadically and had been reported every year in various cities of Punjab, Sindh, and Khyber Pakhtunkhwa provinces [19,20]. A severe dengue outbreak occurred in Lahore district in 2011, during which around 20,000 cases and over 300 causalities were reported [18]. Moreover, the widespread distribution of *Ae. aegypti* caused historic human morbidity and mortality in different parts of Pakistan during the second decade [21,22]. Recent resurgences of vector-borne diseases, including dengue, in Pakistan and concerns of global climate change have together prompted questions regarding their potential relationship [23,24]. For example, increasing evidence of vector-borne disease transmission and associated patterns might be related to anthropogenic climate change [25].

Therefore, the current study was conducted to determine the effects of temperature and relative humidity on egg hatching and adult emergence of the dengue vector *Ae. aegypti.* The influence of population density on the survival of *Ae. aegypti* larvae were also studied. Moreover, we also determined the relationship of maximum temperature (Tmax), relative humidity, and precipitation with the incidence of larval population and inflow of dengue patients during the four-year (2016–2019) study period. The results will be helpful in understanding the relationship between climatic change and vector-borne dengue occurrence and spread. These findings will not only provide a better understanding of abrupt climatic variations and arbovirus vector distribution within Pakistan but also provide support in modulating the strategic approach towards the control of vector populations in developing countries.

## 2. Materials and Methods

### 2.1. Meteorological and Epidemiological Data

**Figure 1:**
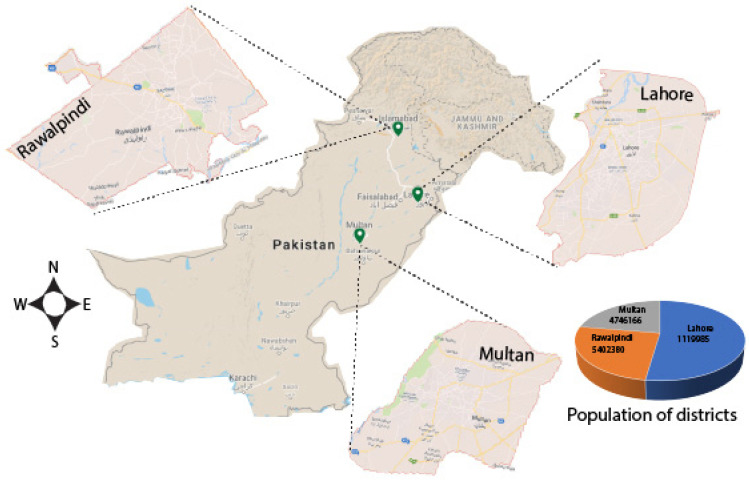
Map of Pakistan with the locations included in this study. The map shows the three districts of Punjab province, Pakistan, i.e., Lahore, Rawalpindi, and Multan. The pie chart represents the total population density of three districts of Punjab province, Pakistan.

The study was conducted in three districts of Punjab, Pakistan, including Lahore, Multan, and Rawalpindi. Among these, the provincial capital city, Lahore (31°32′59″ N 74°20′37″ E) is located in the Northeastern side of Punjab province, Pakistan, with an area of 1772 km^2^ and an estimated population of 11,119,985 inhabitants (Pakistan Bureau of Statistics, 2017, https://www.pbs.gov.pk/content/final-results-census-2017-0, accessed on January 2023), having 6275.39 inhabitants per km^2^. The climate of Lahore is classified as semi-arid according to the Köppen climate classification (Figure 1). Rawalpindi (33°36′ N 73°02′ E) is also located in the Northeastern side of Punjab province, Pakistan, with an area of 5285 km^2^ and an estimated population of 5,402,380 inhabitants (Pakistan Bureau of Statistics, 2017, https://www.pbs.gov.pk/content/final-results-census-2017-0, accessed on January 2023), having 1022.21 inhabitants per km^2^. Rawalpindi’s climate is classified as humid subtropical according to the Köppen climate classification (Figure 1). Multan (30°11′52″ N 71°28′11″ E) is located in the southwestern side of Punjab province, Pakistan, with an area of 3720 km^2^ and an estimated population of 4,746,166 inhabitants (Pakistan Bureau of Statistics, 2017, https://www.pbs.gov.pk/content/final-results-census-2017-0, accessed on January 2023), having 1275.85 inhabitants per km^2^. Its climate is classified as arid according to the Köppen climate classification (Figure 1).

### 2.2. Mosquito Culture and Maintenance

The larval population of *Ae. aegypti* was collected from the district Faisalabad (31°25′0″ N 73°5′28″ E), Punjab, Pakistan. The F1 progeny of the field-collected larval population of *Ae. aegypti* was reared on fish food (Supervit^®^, Tropical, Krausnick-Groß Wasserburg, Germany) in plastic larval trays (10 × 8 × 3 cm^3^). The pupae were transferred to adult cages (30 × 30 × 30 cm^3^) made with a metal frame and nylon net (36 holes/inch^2^), where they became adults within 24 h. The adult population was maintained in the laboratory with continued access to 10% sugar solution and weekly blood feeding on an anesthetized albino rat [26]. The laboratory colony was maintained in the Insect Chemical Ecology Laboratory (ICEL), Department of Entomology, University of Agriculture, Faisalabad, Pakistan, since 2015 at a 27 ± 2 °C temperature, 65–70% relative humidity, and a photoperiod of 12:12 h (D:L).

### 2.3. Effect of Temperature and Humidity

To assess the impact of temperature and relative humidity on the developmental stages of *Ae. aegypti*, four variable levels of temperature (10, 20, 30, and 35 °C) and relative humidity (50, 60, 70, and 80%) were maintained in the insect growth chamber (Memmert, Schwabach, Germany). In total, 16 treatments were included with a combination of four levels of temperature and relative humidity.

One hundred eggs of *Ae. aegypti* were added in an experimental tray (10 × 8 × 3 cm^3^) containing water and placed in an insect growth chamber at the tested temperature and relative humidity. Throughout the experiment, all the developmental stages (i.e., from egg to adult) were observed.

### 2.4. Density-Dependent Trials

First-instar larvae were selected and separated into five groups, i.e., 200, 400, 600, 800, and 1000 larvae per tray containing 1000 mL of water. The population density of each group differed in terms of the number of larvae in each experimental tray (10 × 8 × 3 cm^3^). The larvae were offered fish food (Supervit^®^, Tropical, Germany) at a dose rate of 0.6 mg per larva. Pupae were transferred to the adult cages (30 × 30 × 30 cm^3^) with a 10% sugar solution. The survival rate, mortality, and larval duration was recorded at a constant temperature of 27 ± 2 °C and 65–70% R.H [27].

### 2.5. Data Collection

The meteorological data—monthly rainfall, lowest and highest temperatures, and relative humidity—were provided by the Pakistan Meteorological Department (PMD), Lahore, Pakistan. The average relative humidity (RHavg) was obtained from the morning and evening RHs. In the study period 2016–2019, the data regarding the monthly number of dengue patients and number of *Aedes* larvae were collected from the Directorate of General Health Services, Punjab, Pakistan. Population data were obtained from the latest Census and Statistic Report available at the Pakistan Bureau of Statistics, Pakistan, in 2017 (https://www.pbs.gov.pk/content/final-results-census-2017-0, accessed on January 2023).

### 2.6. Statistical Analyses

Data were statistically analyzed using R software, Version 4.1.2. One-way analysis of variance was used to examine the impact of temperature and relative humidity on egg hatching and adult emergence, as well as on the population density data. Means with significant differences were separated using Tukey–Kramer’s Honestly Significant Difference (HSD). The Pearson correlation coefficient was used to evaluate the relationship among the incidence of larval population, dengue cases, temperature, precipitation, and relative humidity during each month of the study period. The daily value of all *Ae. aegypti* larvae collected within the same district were pooled to yield a single data point on a monthly basis.

The original time series for all three cities were non-stationary. The autocorrelation function (ACF) plots reflect non-stationary time series for all three cities. Different Autoregressive Integrated Moving Average (ARIMA) models were used to make each time series (i.e., for Lahore, Rawalpindi, and Multan cities) stationary. The best model was selected with the minimum AICC (Akaike’s Information Corrected Criterion). The best model chosen based on the minimum AICC was ARIMA (0,0,1)(1,1,0) [17] with drift, ARIMA (0,1,2)(0,1,0) [17], and ARIMA (0,0,1)(0,1,1) [17] with drift for Lahore, Rawalpindi and Multan, respectively.

## 3. Results

### 3.1. Egg Hatching and Adult Emergence

A reduction in egg hatching and adult emergence was observed with the changing temperature gradient and relative humidity, whereas the intensity of the reduction was not influenced by the relative humidity at extreme temperatures (10 and 35 °C). The temperature and RH were interlinked and had a significant impact on the developmental stages of *Ae. aegypti* at propitious temperature and relative humidity regimes (df = 16, F = 31.87, *p* ≤ 0.0001). The results further showed that that average mosquito survival at 30 °C was significantly highest at 50% RH (*p* ≤ 0.0001).

**Figure 2:**
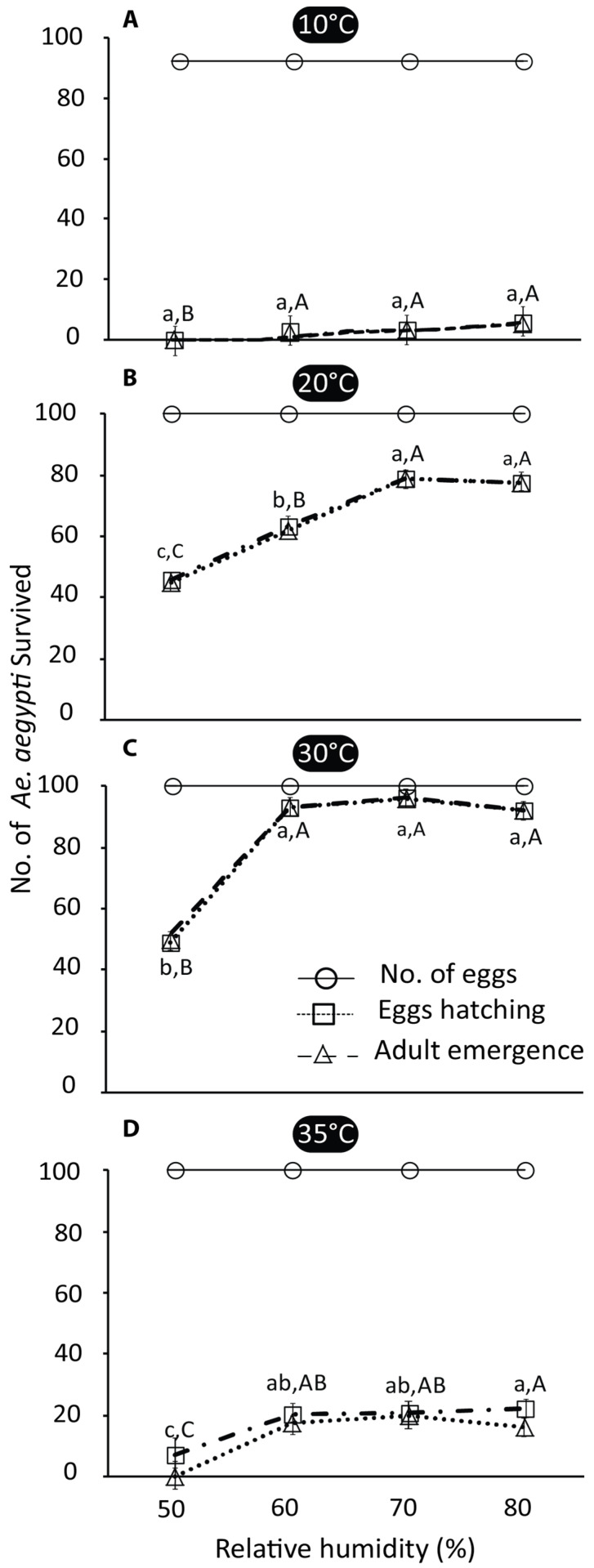
Behavioral response of *Ae. aegypti* on egg hatching and adult emergence with various temperature and relative humidity regimes (N = 3) was examined using one-way ANOVA (HSD test). (**A**), (**B**), (**C**), and (**D**) denote the survival of *Ae. aegypti* larvae and adults at different temperatures, i.e., 10 °C, 20 °C, 30 °C, and 35 °C, respectively. Letters denote the significant difference over increasing relative humidity levels at each temperature. Relative humidity and the number of *Ae. aegypti* are shown in the *x*-axis and *y*-axis, respectively. Error bars signify the standard error mean (SEM).

The results further showed that at 10 °C, egg hatching and adult emergence significantly reduced to 2.6 ± 0.33 and 2.3 ± 0.33 at 60% RH, 3.33 ± 0.33 and 3.33 ± 0.33 at 70% RH, and 5.3 ± 0.33 and 5 ± 0.57 at 80% RH, respectively (for egg hatching: df = 3, F = 58.22, *p* ≤ 0.00; for adult emergence: df = 3, F = 31.47, *p* ≤ 0.00). However, at 50% RH, no egg hatching was observed (Figure 2A). Similarly, at 20 °C, there was a significant reduction in egg hatching and adult emergence at 50% RH (46 ± 2.08 and 45 ± 2.64) compared to 60, 70, and 80% RHs. However, egg hatching and adult emergence significantly increased at 70 and 80% RHs (egg hatching: 78.66 ± 1.85, 78.66 ± 1.85; adult emergence: 77.66 ± 1.45, 77.66 ± 1.45) compared to 60% RH (egg hatching: 63.33 ± 1.76; adult emergence: 62 ± 2.08), respectively (egg hatching: df = 3, F = 72.10, *p* ≤ 0.00; adult emergence, df = 3, F = 59.48, *p* ≤ 0.00) (Figure 2B).

The results further depicted that at a temperature of 30 °C and 60, 70, and 80% RHs regimes, the recorded egg hatching was 93 ± 1.15, 96 ± 0.88, and 92 ± 1.52 eggs, while the reported adult emergence was 93 ± 1.15, 95 ± 0.66, and 92 ± 1.52, respectively. Egg hatching and adult emergence showed a non-significant effect at all higher RH regimes, which differed significantly from 50% RH, with mean values of 48 ± 0.57 and 47 ± 0.92 (egg hatching: df = 3, F = 441.49, *p* ≤ 0.000; adult emergence: df = 3, F = 4.69.10, *p* ≤ 0.000) (Figure 2C). Similarly, at 35 °C and 60, 70, and 80%, significant differences were observed regarding egg hatching—20 ± 0.33, 20 ± 1.45, and 22 ± 2.33—and adult emergence—17 ± 0.33, 20 ± 1.15, and 16 ± 2—when compared with 50% RH, with mean values for egg hatching of 7 ± 1.52 and adult emergence of 0 ± 0.00 (egg hatching: df = 3, F = 20.22, *p* ≤ 0.00; adult emergence: df = 3, F = 60.08, *p* ≤ 0.00) (Figure 2D). Comparative analysis revealed that egg hatching and adult emergence were significantly affected by lower and higher temperatures (10, 35 °C) regardless of the relative humidity (df = 3, F = 617.24, *p* ≤ 0.00) (Figure 2D).

### 3.2. Larval Density

**Figure 3:**
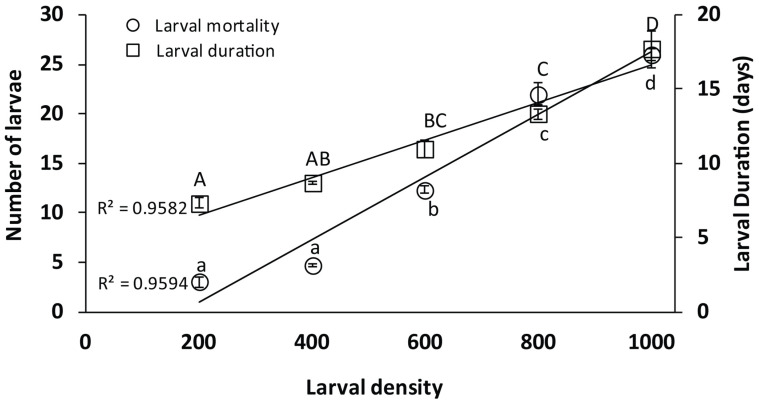
Survival of *Ae. aegypti* at various population density levels (N = 3) was examined using one-way ANOVA (HSD test). The right axis represents the larval duration, and the left axis denotes the number of larval mortalities. Letters denote the significance level at an increasing density of mosquitoes. Error bars signify the standard error mean (SEM). R^2^ represents the regression value.

The larval duration and mortality at a constant temperature (27 ± 2 °C) and RH (65–70 ± 2%) were significantly higher with increased larval densities (df = 4, F = 308.43, *p* ≤ 0.00). Similarly, the larval duration period was significantly prolonged with increased larval numbers (df = 4, F = 53.82, *p* ≤ 0.00) (Figure 3). The linear regression line for larval mortality (R^2^ = 0.9594) and larval duration (R^2^ = 0.9582) indicated that the number of larval populations below 600 has no significant effect on the larval mortality.

### 3.3. Meteorological Data

**Figure 4:**
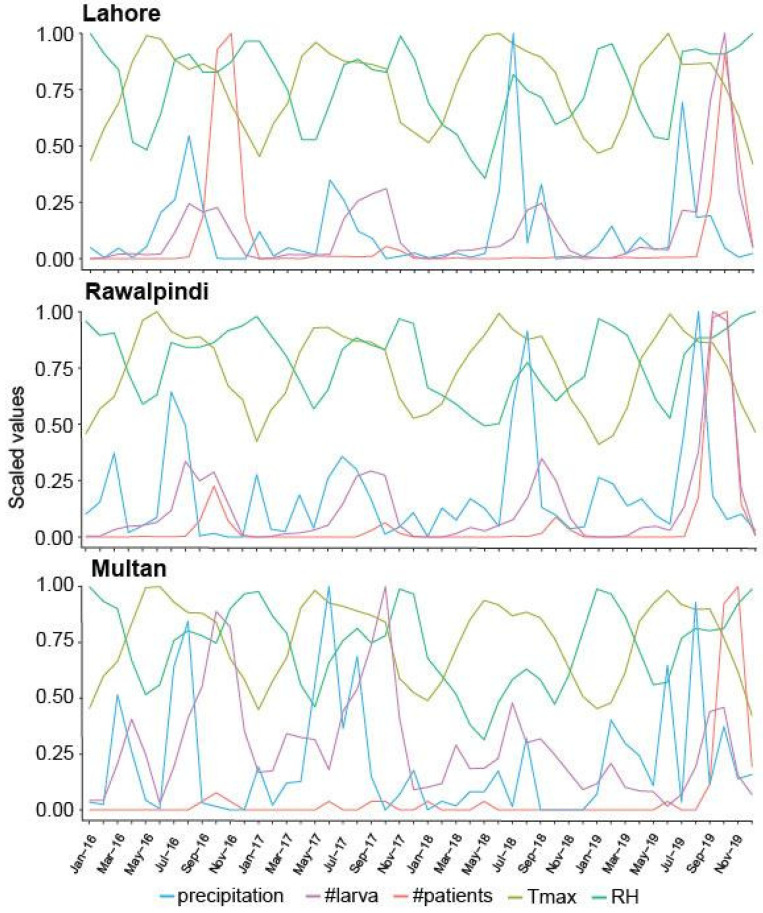
Incidence of *Ae. aegypti* larvae and number of dengue patients in relation to temperature, relative humidity, and precipitation throughout the reporting years 2016–2019 for three studied districts of Punjab province, Pakistan, using Pearson’s correlation coefficient. The *y*-axis represents the scale value of each variable that is presented with different color lines. The *x*-axis denotes the months of each year (2016–2019).

The monthly temperatures of Lahore, Rawalpindi, and Multan districts varied between 8.1 and 40.6 °C, 7.7 and 38.7 °C, and 8.4 and 42.1 °C, respectively (Table 1 and Figure 4). Higher variations in the monthly RH of Lahore, Rawalpindi, and Multan districts were found to be 17.4–87, 19–95, and 18.2–91%, respectively (Table 1 and Figure 4). Meanwhile, the monthly rainfall patterns for Lahore, Rawalpindi, and Multan districts were 115.5–577.5 mm, 107.1–535.5 mm, and 16.34–81.7 mm, respectively (Table 1 and Figure 4). The highest rainfall occurred from May to September, while its pattern was infrequent for the rest of the year (Table 1 and Figure 4). The meteorological data were observed for the study period from 2016 to 2019.

### 3.4. Temporal Occurrence of Vector Aedes Aegypti Larval Population

The population dynamics trends of *Ae. aegypti* larvae were observed from three epidemic districts—Lahore, Rawalpindi, and Multan—of Punjab province in Pakistan for each month throughout the study period 2016–2019 (Figure 4). The data depicted that the population of vector larvae is less than 600 in all the reported districts during the winter season (December, January, and February), whereas the vector occurrence was less than 2000 during the spring and summer seasons (March, April, May, and June) (Figure 4). However, a gradual increase in the epidemic vector population occurred during the autumn season (July, August, September, October, and November), where this projectile population dynamic begins in July and falls in November (Figure 4). The vector population trend was almost the same, below a 0.3 scale value, in Lahore and Rawalpindi districts in 2016, 2017, and 2018 (Figure 4), whereas the vector population trend increased by 1.0 scale value in both districts in 2019 during the autumn season. However, there was no significant change in the vector population in Multan district throughout the study period compared to Lahore and Rawalpindi districts (Figure 4). Moreover, the incidence of *Ae. aegypti* larvae was directly proportion to the pattern of rainfall with the lag of 1–2 months during the study period (Figure 4). An association between rainfall and larval emergence was observed, i.e., when rainfall > 200 mm occurred, the larvae emerged as shown in Lahore and Rawalpindi districts (Figure 4).

### 3.5. Pearson Correlation Analysis

**Figure 5:**
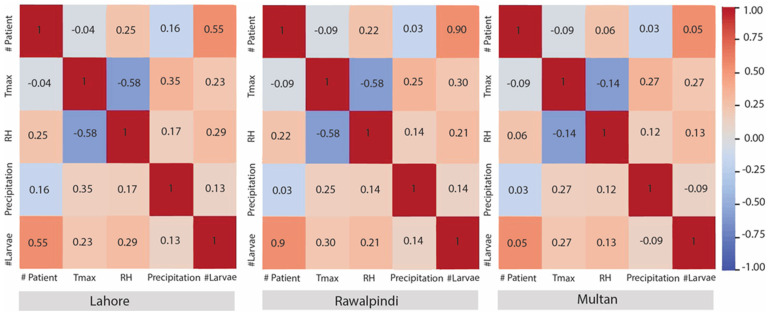
A heat map presenting the results of a Pearson correlation coefficient analysis association between temperature, RH, precipitation, larval incidence, and the number of patients in three districts of Punjab province, Pakistan, while scaled with its maximum value during the studied years. The color gradient represents the different levels of correlation among each variable. In addition, color shading relative to specific correlation values are not directly comparable between study localities.

**Figure 6:**
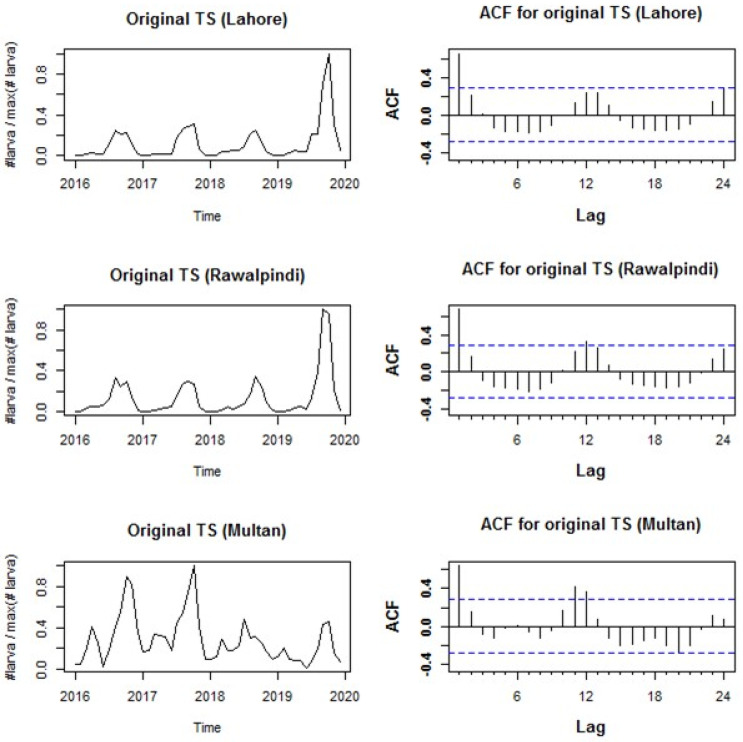
The original time series (**left column**) and the corresponding autocorrelation functions (ACFs) (**right column**) are plotted for each district. In the left column, the *x*-axis depicts the number of years in the time series, while the *y*-axis represents the number of larvae per maximum number of larvae in the scaled value for each district. In the right column, the *x*-axis shows the number of lag months, and the ACF scaled value is depicted in the *y*-axis.

**Figure 7:**
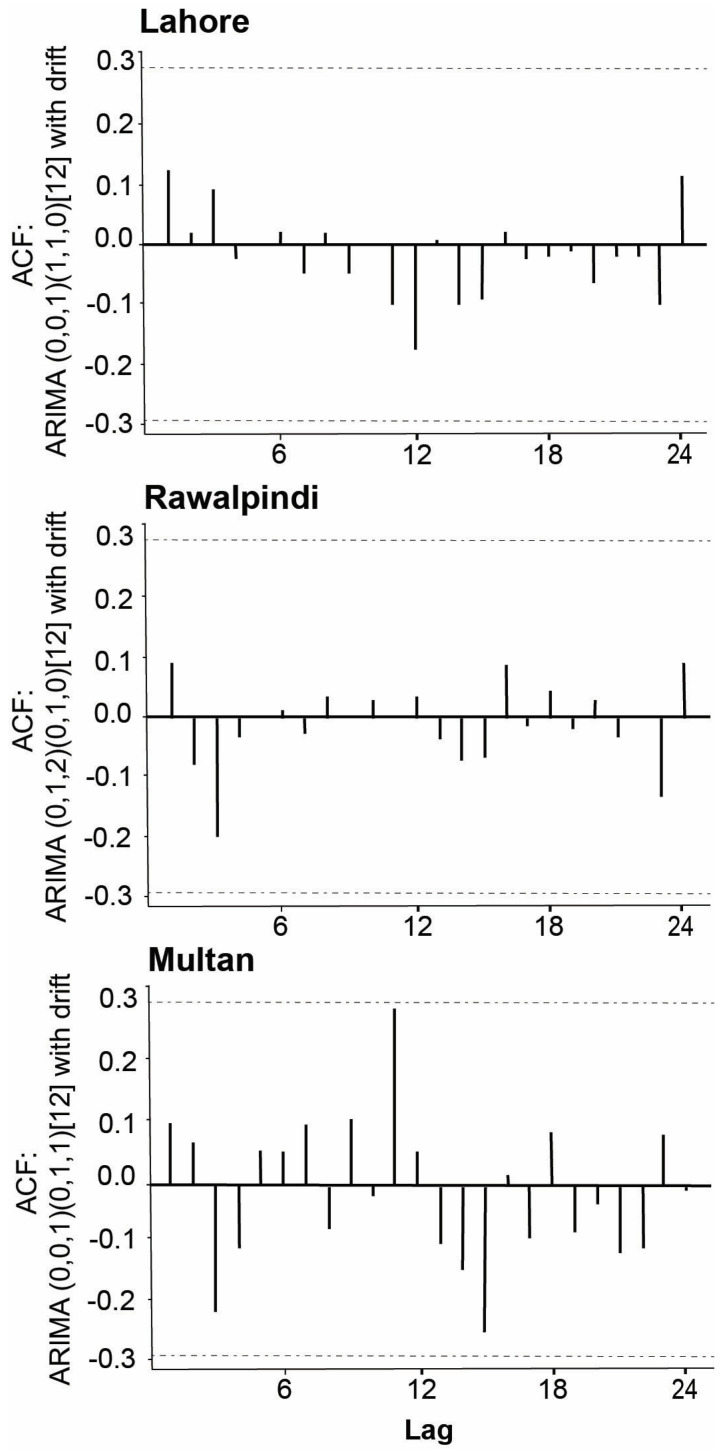
ACF plots for the residuals obtained from the ARIMA fitted models to the time series for Lahore, Rawalpindi, and Multan districts. The *x*-axis represents the lag time in months, and the *y*-axis shows the ACF with the ARIMA fitted model.

To determine the level of association of various parameters, i.e., Tmax, RH, and precipitation, with the larval incidence and number of dengue patients, a Pearson correlation coefficient analysis was performed. The Pearson correlation analysis revealed that larval incidence was positively correlated with number of dengue patients, Tmax, RH, and precipitation at Lahore (0.55, 0.23, 0.29, and 0.13), Rawalpindi (0.90, 0.30, 0.21, and 0.14), and Multan (0.05, 0.27, and 0.13), respectively, except in Multan, where a negative correlation (−0.09) with precipitation was observed (Figure 5). However, the number of dengue patients had a positive correlation with relative humidity at Lahore (0.25), Rawalpindi (0.22), and Multan (0.06). In addition, it also had a positive correlation with precipitation at Lahore (0.16), Rawalpindi (0.03), and Multan (0.03) (Figure 5) with a lag of 1–2 months (Figure 6 and Figure 7). In contrast, a negative correlation was observed with Tmax of −0.04, −0.09, and −0.09 at Lahore, Rawalpindi, and Multan, respectively (Figure 5). Figure 5 depicted that the 1–2-month lag was consistent, i.e., precipitation > larval occurrence > dengue patients, each year in all the studied districts. However, the Tmax and RH have a negative correlation in Lahore, Rawalpindi, and Multan, at −0.58, −0.58, and −0.14, respectively.

Figure 6 (left column) showed a strong seasonal pattern of larval incidence with the temporal variations during the study period, with the spikes occurring around the autumn period of all the years. Similar spikes were observed in several patients with a lag of 1–2 months (Figure 4 and Figure 6; right column). In contrast, a 1–2-month lag was observed while the incidence of larvae after precipitation (Figure 4 and Figure 7). Predictive ARIMA models were used previously to forecast the burden of dengue fever cases, while the same model was used to predict the occurrence of larvae in this study. Four years of monthly larval incidence data from three districts of Punjab province in Pakistan were used in the current study. Seasonal ARIMA (0,0,1)(1,1,0) [17] with drift, ARIMA (0,1,2)(0,1,0) [17], and ARIMA (0,0,1)(0,1,1) [17] with drift for Lahore, Rawalpindi, and Multan, respectively, were selected as the best-suited models to predict the future incidence of larvae in the upcoming year.

**Figure 8:**
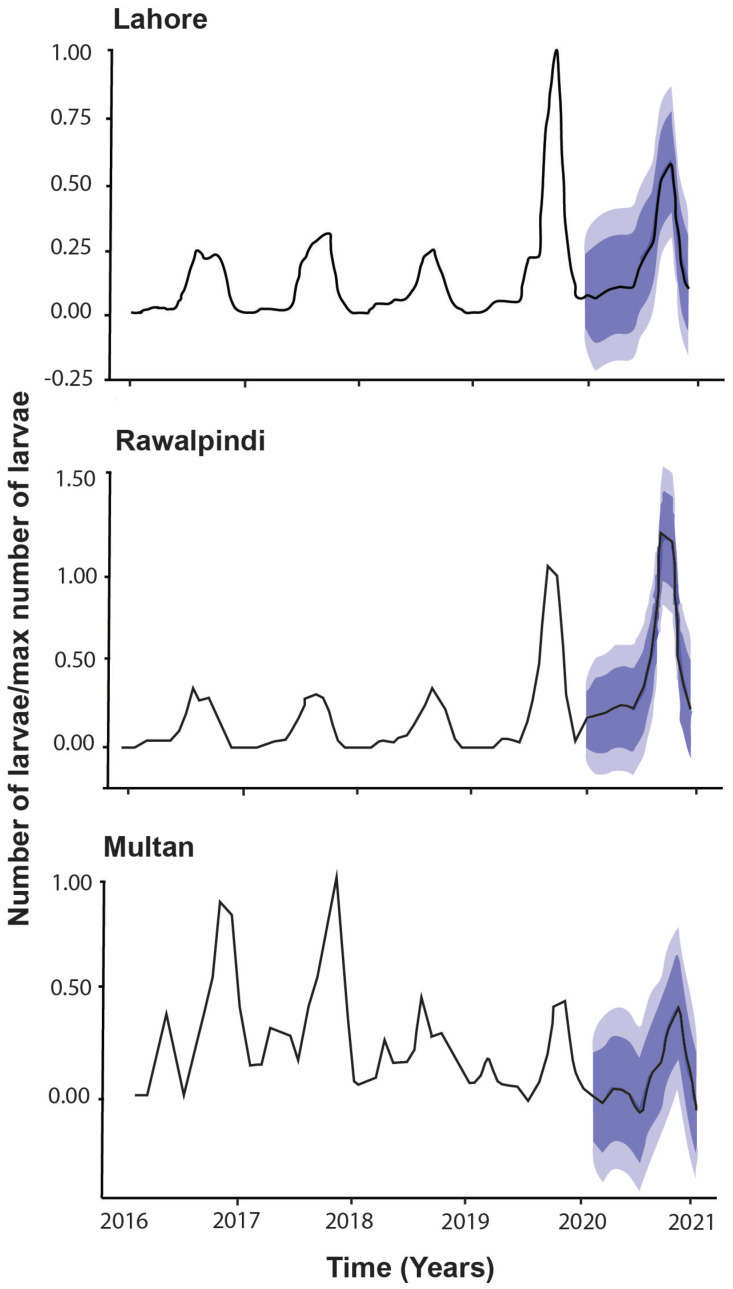
Forecasts for different districts using the best ARIMA fitted model. The line shows the point forecast. The shaded lines represent the predicted scaled values of larval incidence in the year 2020. The dark grey represents the 80% CI, and light grey is showing the 95% CI for the forecasts.

The ACF plots of the residuals from the models ARIMA (0,0,1)(1,1,0) [17] with drift, ARIMA (0,1,2)(0,1,0) [17], and ARIMA (0,0,1)(0,1,1) [17] with drift for Lahore, Rawalpindi, and Multan, respectively, showed that all autocorrelations lie between the threshold limits. The resulting ACF plots reflected that the residuals were white noise for the fitted models to the time series of different cities under the study period. A portmanteau test also provided the *p*-values 0.98, 0.94, and 0.60 for Lahore, Rawalpindi, and Multan time series, respectively. The large *p*-values also suggested that the residuals were white noise in each fitted model (Figure 6 and Figure 7). The forecasts regarding the number of larvae in each district were predicted with the help of the most appropriate ARIMA fitted model, and the results of the point forecast with their 80% and 95% CIs are shown in Figure 8. The predicted occurrence of larvae in 2020 was expected to be higher in Rawalpindi compared to Lahore, followed by Multan (Figure 8).

## 4. Discussion

We sought to estimate the larval survival of *Ae. aegypti* across different regimes of environmental factors, such as temperature, relative humidity, and precipitation, and hypothesized that these factors would influence the survival of *Ae. aegypti* larvae as well as the incidence of dengue patients in three districts (Lahore, Rawalpindi, and Multan) of Punjab, Pakistan. Our study provided data to support the association between variations in abiotic factors and the occurrence of *Aedes* larvae that were linked to the incidence of dengue patients. Thus, the climatic variations, including temperature, relative humidity, and precipitation, had an impact on the density of *Ae. aegypti.* The current results were further validated by numerous studies, which reported that temperature, humidity, wind, and rainfall are determinant factors that can affect the oviposition, viability of eggs, larval development, longevity, and adult dispersion of dengue vectors [28,29,30].

### 4.1. Effects of Temperature and Relative Humidity

The current results indicated that *Ae. aegypti* egg hatching and adult emergence were correlated with the temperature and relative humidity. There was a significant effect of a gradual increase in the temperature and relative humidity on the eggs hatching and adult emergence, which is in line with the previous findings [31,32]. However, the production of eggs was also dependent on the temperature and humidity [19], which revealed that higher egg hatching and adult emergence were observed at 20 °C and 70 and 80% RHs. The inverse was found at 35 °C and 50, 60, 70, and 80% RHs, where the number of eggs that hatched was significantly reduced regardless of the RH. These results are in line with the findings of Mohammed and Chadee [33], who reported that an increase in the temperature significantly decreased the hatching rates of *Ae. aegypti* from 98% at 24–25 °C to 2% at 34–35 °C. Furthermore, high temperatures can also affect the survival of *Ae. aegypti* immatures [34]. In addition, at an extremely low temperature (10 °C), egg hatching was significantly reduced and ultimately reduced the adult emergence, which correlates with the findings that the proportion of eggs hatching of *Ae. aegypti* was significantly reduced below 14 °C [35,36]. The hatch rate was significantly lower at 37 °C (57%), and no eggs hatched at 40 °C. The hatching of *Ae. aegypti* eggs is typically influenced by the RH. An increased RH usually enhances egg hatching rates probably because of lower egg desiccation. The increased humidity mitigates water loss from the eggs, allowing them to remain viable and develop for a longer period of time [37,38].

Another study showed that eggs do not survive at high temperatures, i.e., 43–44 °C [39], and larvae at low (10–12 °C) and high temperatures (38–40 °C) [40]. A previous study reported that moderate temperature (15–27 °C) and a higher RH (55–75%) positively impact egg hatching percentages, whereas at 32 and 35 °C, the percentage of eggs hatching was decreased [41], as evident in our study. However, no positive effect of RH alone has been observed on *Aedes* mosquitos’ activity [42], which is also in line with our findings that variable temperature regimes significantly reduced egg hatching and adult emergence at a level of 50% RH. The results of the current study are also associated with previous findings, in which reduced fecundity and longevity, adult mortality, and oviposition have been related to a rise in temperature and humidity [8,23]. In addition, egg hatching of *Anopheles albimanus* significantly increased at 30 °C compared to 25 °C [43]. These findings propose that the variation in the population of *Ae. aegypti* throughout the year may be prejudiced by the effect of high temperatures and low humidity. Our study suggests that in the future, climate change might further enhance the global temperature, which may influence egg hatching and adult emergence. In line with the recent findings, the increased temperature may cause reductions in the vectorial capacity and development period of *Aedes* mosquitoes [44,45].

### 4.2. Density-Dependent Trials

The impact of larval densities on the survival of *Ae. aegypti* larvae was examined in the laboratory under a constant temperature and RH. To avoid larval competition for food, a measured amount of larval diet was provided [27]. In this study, the average proportion of larval mortality in the two highest larval density treatments (800 and 1000 larvae) was significantly higher compared to three lower larval densities. However, the larval duration also increased with an increase in larval densities [46]. Our findings suggested that the larval mortality and duration were directly proportional to high larval densities under a controlled temperature and relative humidity, which correlates with previous findings where larval mortality was interlinked with the larval densities under diurnal temperature patterns [47,48].

### 4.3. Temporal Occurrence of Vector Population

This study represents the seasonal variations in temperature, RH, and precipitation patterns, as well as their correlation with the vector larvae and dengue patient incidence. The current findings represent a positive correlation between the monthly incidence of larvae and monthly maximum temperature, RH, number of patients, and precipitation. In this study, during the winter season, a lower temperature (<20 °C), higher RH (≥80%), and precipitation (<100 mm) resulted in a lower incidence of the larval population (0.00 scale value); however, a higher temperature (>32 °C), RH (≤70%) and precipitation (<100 mm) during spring and summer seasons resulted in a lower incidence of the larval population (<0.1 scale value). These findings are in line with previous thoughts that the temperature range <18 °C and >35 °C can hinder the incidence of larval populations and dengue transmission [42]. Another study demonstrates that temperature significantly affects the developmental stages, mosquito size, feeding capacity, and fecundity of the *Culex* mosquitoes. Moreover, temperatures more than 30 °C may lead to enhanced mosquito mortality [49].

Interestingly, during the autumn season, an optimum temperature (>30 °C), RH (~70%), and precipitation (~300 mm) resulted in a higher occurrence of larval population and incidence of dengue patients (<0.3 scale value) during the study period from 2016–18 and <1.00 in 2019 in all the districts. A significant correlation was observed between monthly rainfall and larval incidence compared to maximum temperature and RH, which is in line with previous findings, where a positive correlation was observed between rainfall and the incidence of malaria [50,51] and distribution of dengue in Taiwan [52]. Similarly, a positive association between dengue incidence, relative humidity, and precipitation, recorded in Lahore (0.25, 0.16) Rawalpindi (0.22, 0.03), and Multan (0.06, 0.03), was correlated with previous studies [52,53]. In contrast, dengue incidence has a negative correlation with the maximum temperature, which has been previously reported in Sri Lanka [53], and in our findings, a higher temperature reduced the survival rate and vectorial capacity of *Aedes* mosquitoes [39] and development period [40].

The incidence of larvae and dengue cases was enhanced during the autumn season in reported districts of Pakistan. This was also confirmed using the ARIMA model autocorrelation analysis that rainfall before a 1–2-month lag period could be responsible for the outbreak of larvae and dengue incidence in the reported districts. Our findings from autocorrelation analysis correlate with previous studies that depict a 4–8-week lag time between rainfall and dengue incidence in Sri Lanka and Port Sudan City [54,55]. However, occasional dengue cases may result due to irregular rainfall, water storage practices, and indoor manmade breeding sites that were reported in Southeast Asian countries like Indonesia, Singapore, and Thailand [56]. The lower number of dengue cases in Multan district could be due to a high average temperature (>30 °C), low humidity, and low precipitation (<80 mm) resulting in fewer larval incidences (1000), as well as less population density, compared to Lahore and Rawalpindi throughout the year. However, the present study points out the association between rainfall and dengue patient incidence related to 1–2 months of prior rainfall. As per our understanding, a level of 1–2-month lag association follows this pattern, i.e., precipitation > larval occurrence > dengue patient incidence.

**Figure 9:**
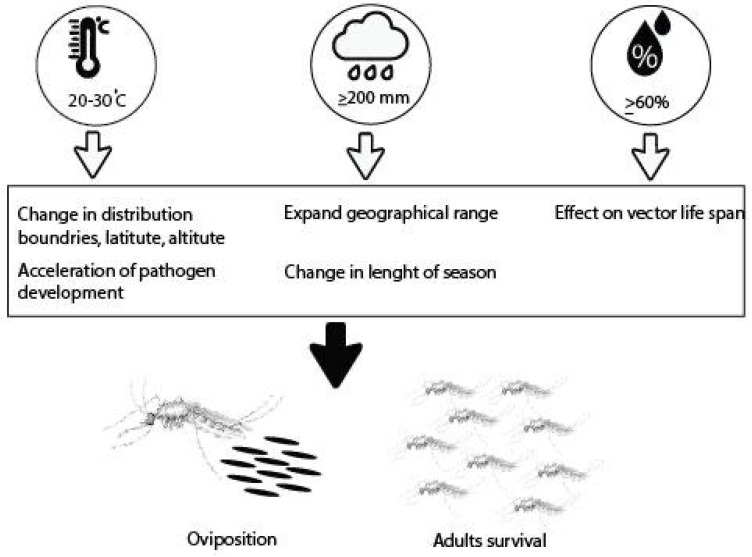
Schematic layout of optimal abiotic parameters influences the number of larvae and dengue patient incidence.

Predictive ARIMA models were used previously to forecast the burden of dengue fever cases, while the same model was used to predict the occurrence of larvae, which shows the incidence of larvae in the upcoming year depending on environmental factors, which will be higher in Rawalpindi district than the previous years, followed by Lahore and Multan. Similar findings were also reported, where it was found that rainfall, relative humidity, and temperature significantly affected dengue occurrence in East Delhi [57] Malaysia [58] and Singapore [56]. The schematic layout of optimal parameters is depicted in Figure 9.

### 4.4. Population Density

Lahore and Rawalpindi districts have been undergoing a very high dengue incidence and the highest number of cases reported during the study period. The two major reasons behind these higher numbers of reported cases in Lahore and Rawalpindi were that (1) the rainfall pattern was optimal, with a favorable temperature and humidity for the survival and development of *Aedes* mosquitoes during the autumn season, and (2) the population density was higher in these districts compared to Multan district. A similar observation has been reported in Sri Lanka, where a higher number of dengue cases were reported in highly populated districts like Jaffna, Batticaloa, and Colombo, possibly due to frequent traveling at the end of the war in 2009–2010 [59,60]. Hence, the increased population density and precipitation pattern may elevate the incidence of larvae and dengue cases in Pakistan.

### 4.5. Climate Change

In the current climatic shifts, a change in global atmospheric temperature and rainfall patterns has had profound impacts on the abundance of *Ae. aegypti* population dynamics, geographical shifts, and vector-borne diseases spread by *Ae. aegypti* around the globe [58]. *Aedes aegypti* distribution is highly vibrant in space and time, as its life cycle is short and severely influenced by environmental variations [59]. Previous studies have shown a correlation between temperature and the abundance of female mosquitoes [60] and found that *Ae. Aegypti*’s ability to transmit dengue virus (DENV) is temperature dependent [61,62]. Similarly, it has also been observed that the time of virus detection in the salivary gland decreases from 9 to 5 days while feeding at 26 and 30 °C, respectively, for DENV-1 and DENV-4 [63]. Moreover, a high temperature, not more than 35 °C (current study), may enhance the rate of blood feeding and decrease the extrinsic incubation period [64,65]. So, it can be concluded that climate change may expand the dispersal of *Ae. aegypti* and dengue virus to previously dengue-free areas [66] and is expected to spread towards moderate climate due to an increase in the temperature, as suggested that the suitable ecological niche for *Aedes* will expand with climate change in Canada and the United States [35].

## 5. Conclusions

This study demonstrates the association of climatic factors linked with larval occurrence and dengue incidence. The results described the role of precipitation > 200 mm prior to 1–2 months of lag, 20–30 °C, and above 60% RH, which lead to the occurrence of larvae and dengue cases spiking if there is no management adapted earlier. This study will help to reinforce dengue surveillance and control strategies in Pakistan and to establish early management strategies based on climate factors and research. Health authorities must synchronize national surveillance systems with research institutes and the meteorological department for the development of an integrated approach for mosquito management, as climate change could lead to a shift in mosquito’s abundance to favorable climate zones of the country.

## Figures and Tables

**Table 1:** Data represent the variable ranges of Tmax, relative humidity, precipitation, number of larvae, and number of patients against each scale value for Lahore, Rawalpindi, and Multan districts of Punjab province in Pakistan during the study period.

Lahore					
Scale Value	No. of Patients	Tmax	RH	Precipitation	No. of Larva
0	0	0	0	0	0
0.2	95	8.12	17.4	115.5	17,881
0.4	190	16.24	34.8	231	35,762
0.6	285	24.36	52.2	346.5	53,643
0.8	380	32.48	69.6	462	71,524
1	475	40.6	87	577.5	89,405
**Rawalpindi**					
**Scale Value**	**No. of Patients**	**Tmax**	**RH**	**Precipitation**	**No. of Larva**
0	0	0	0	0	0
0.2	577.6	7.74	19	107.1	11,869
0.4	1155.2	15.48	38	214.2	23,738
0.6	1732.8	23.22	57	321.3	35,607
0.8	2310.4	30.96	76	428.4	47,476
1	2888	38.7	95	535.5	59,345
**Multan**					
**Scale Value**	**No. of Patients**	**Tmax**	**RH**	**Precipitation**	**No. of Larva**
0	0	0	0	0	0
0.2	5.2	8.42	18.2	16.34	224.6
0.4	10.4	16.84	36.4	32.68	449.2
0.6	15.6	25.26	54.6	49.02	673.8
0.8	20.8	33.68	72.8	65.36	898.4
1	26	42.1	91	81.7	1123

## Data Availability

The original contributions presented in this study are included in the article. Further inquiries can be directed to the corresponding authors.
